# Promoting research quality

**DOI:** 10.1371/journal.pbio.3002554

**Published:** 2024-02-27

**Authors:** Ulrich Dirnagl, Nonia Pariente

**Affiliations:** 1 BIH QUEST Center for Responsible Research, Berlin Institute of Health at Charité (BIH), Berlin, Germany; 2 Public Library of Science, San Francisco, California, United States of America, Public Library of Science, Cambridge, United Kingdom

## Abstract

Plenty of awards recognize scientific contributions, but a unique and important one honors those whose efforts significantly enhance the quality and robustness of research. This Editorial discusses why this is important to promote trust in science and the need to reform how we assess research/researchers.

## Teaser

Plenty of awards recognize scientific contributions, but a unique and important one honors those whose efforts significantly enhance the quality and robustness of research. We discuss why this is important to promote trust in science.

Researchers around the globe invest tremendous effort to promote societal progress and offer guidance in navigating a complex world. New knowledge builds on the foundation of existing knowledge and previous research and, like a house, is only as good and durable as its foundation. This is true for biology and medicine [1] just as much as for historical sciences or physics. Only evidence-based research conducted with rigor, using reliable, robust methodology, and transparent reporting enables comprehensive and significant scientific advance.

However, there are often substantial weaknesses in planning, conducting, analyzing, and reporting research. Low internal and external validity, as well as low statistical power, produces a high rate of false positives and inflates effect sizes unrealistically. Unsurprisingly, recent large-scale reproducibility initiatives have shown that key findings of highly cited studies often cannot be reproduced, and replications only succeed with dramatically shrunken effect sizes [2]. On the other hand, nonpublication of results leads to duplicative research and deprives decision makers of the totality of evidence. The immense proliferation of publication venues (which is to a large extent a response to volume-based research/researcher assessment processes), combined with the increasing size of datasets and methodological complexity, greatly complicates the sharing, evaluation, and synthesis of high-quality evidence. At the same time, the pressure to publish and secure funding has become so intense that it increasingly compromises research quality. There has been a marked growth of data manipulation and even outright fraud; predatory publishers and paper mills, increasingly supported by artificial intelligence, are flooding the literature with substandard or entirely made-up studies.

Against this backdrop, the Einstein Foundation Berlin is dedicated to honoring those whose efforts significantly enhance the quality and robustness of research findings. There are plenty of prestigious prizes worldwide to honor those who make specific scientific discoveries and contributions, but shining a light on the way research is performed, and rewarding rigor, reliability, robustness, and transparency is crucial. If we do not signal that these things are important, they are at risk of falling by the wayside of the publish or perish machine. Promoting trustworthy research is a collective responsibility; science is not inherently trustworthy and trust must be actively gained/retained. This is crucial if society is to embrace the advances that science may afford. In addition, most research worldwide is funded by the public, and public support is not something that should be assumed but rather nurtured. Too much hangs in the balance.

The Einstein Foundation Award for Promoting Quality in Research recognizes contributions that fundamentally increase the validity of research and science, thereby significantly promoting their societal benefit. Award-worthy activities may, for example, enable worldwide free access to research (Open Science); establish equitable international collaborations (big team science); create amenable structural conditions that shape careers towards high-quality research; or foster transparent dissemination of robust results or the commitment to high ethical standards in research. The Award is funded by the Damp Foundation for a period of 10 years and is also supported by the State of Berlin. The QUEST Center for Responsible Research of the Berlin Institute for Health, Springer Nature, PLOS, the Max Planck Support Foundation, and the Berlin University Alliance all assist the Einstein Foundation Berlin in the international establishment and recognition of the prize. The Award is endowed with a total of €500,000 a year, divided into 3 categories: the “Individual Award” honors individual researchers or small research groups, the “Institutional Award” recognizes organizations and scientific institutions, and the “Early Career Award” rewards innovative ideas from scientists at the beginning of their careers. The 2023 recipients, chosen by an internationally renowned jury, will receive their prizes in an award ceremony in Berlin in March. They were Yves Moreau in the individual category, the “Berkeley Initiative for Transparency in Social Sciences” in the institutional category, and the “Responsible Research Assessment” project led by Anne Gärtner in the early career researcher category.

In this issue of *PLOS Biology*, award-winner Anne Gärtner and 2 colleagues from the Responsible Research Assessment project discuss the broader issue of research assessment reform and their project [3], which aims to identify and test novel criteria for the assessment of researchers and their output. Moving away from volume of output and other unsuitable metrics, the project aims to provide a measurement of research quality by taking into account factors such as transparency, robustness, innovation, and cooperation.

Research assessment is something we have long believed needs urgent reform. For a system largely built on public money, funding agencies and institutions normally assess researchers on the basis of metrics that have little to do with how much return on investment (that is, societal benefit) will come from the research. Considerations such as how robust the results are or how quickly other people will be able to build on the research are not part of this equation. Contributions other than scientific articles are seldom taken into account, and the number of publications is often more important than their quality. This is not a new problem and there are ongoing attempts to change the status quo, such as DORA, CoARA (see the CoARA commitments in Box 1), or the Hong Kong declaration for research assessment [4]. There are also difficulties in moving away from easy-to-use, if flawed, metrics. We welcome the development of new initiatives in this space and look forward to a future in which a different system of research/researcher assessment becomes mainstream.

**Box 1. The CoARA commitments for research assessment reform**

1. Recognize the diversity of contributions to, and careers in, research in accordance with the needs and nature of the research.
2. Base research assessment primarily on qualitative evaluation for which peer review is central, supported by responsible use of quantitative indicators.
3. Abandon inappropriate uses in research assessment of journal- and publication-based metrics, in particular, inappropriate uses of Journal Impact Factor (JIF) and *h*-index.
4. Avoid the use of rankings of research organizations in research assessment.
5. Commit resources to reforming research assessment as is needed to achieve the organizational changes committed to.
6. Review and develop research assessment criteria, tools, and processes.
7. Raise awareness of research assessment reform and provide transparent communication, guidance, and training on assessment criteria and processes as well as their use.
8. Exchange practices and experiences to enable mutual learning within and beyond the Coalition.
9. Communicate progress made on adherence to the Principles and implementation of the Commitments.
10. Evaluate practices, criteria, and tools based on solid evidence and the state-of-the-art in research on research, and make data openly available for evidence gathering and research.
